# Effect of Varying Amount of Polyethylene Glycol (PEG-600) and 3-Aminopropyltriethoxysilane on the Properties of Chitosan based Reverse Osmosis Membranes

**DOI:** 10.3390/ijms22052290

**Published:** 2021-02-25

**Authors:** Anum Kayani, Muhammad Asim Raza, Arsalan Raza, Tajamal Hussain, Muhammad Sarfraz Akram, Aneela Sabir, Atif Islam, Bilal Haider, Rafi Ullah Khan, Sang Hyun Park

**Affiliations:** 1 Institute of Chemistry, University of the Punjab, Lahore 54590, Pakistan; anumkayani57@gmail.com (A.K.); arsalanraza0022@gmail.com (A.R.); tajamalhussain.chem@pu.edu.pk (T.H.); 2 Institute of Polymer and Textile Engineering, University of the Punjab, Lahore 54590, Pakistan; aneela.pet.ceet@pu.edu.pk (A.S.); dratifislam@gmail.com (A.I.); rkhan.icet@pu.edu.pk (R.U.K.); 3 Advanced Radiation Technology Institute (ARTI), Korea Atomic Energy Research Institute, Jeongeup 56212, Korea; mohammadasimraza@yahoo.com; 4 Radiation Science and Technology, University of Science and Technology, Daejeon 34113, Korea; 5 Institute of Energy and Environmental Engineering, University of the Punjab, Lahore 54590, Pakistan; msakram.ieee@pu.edu.pk; 6 Institute of Chemical Engineering and Technology, University of the Punjab, Lahore 54590, Pakistan; bilal.icet@pu.edu.pk

**Keywords:** films, hydrophilicity, surface morphology, cross-linking, permeate flux

## Abstract

Chitosan and polyethylene glycol (PEG-600) membranes were synthesized and crosslinked with 3-aminopropyltriethoxysilane (APTES). The main purpose of this research work is to synthesize RO membranes which can be used to provide desalinated water for drinking, industrial and agricultural purposes. Hydrogen bonding between chitosan and PEG was confirmed by displacement of the hydroxyl absorption peak at 3237 cm^−1^ in pure chitosan to lower values in crosslinked membranes by using FTIR. Dynamic mechanical analysis revealed that PEG lowers Tg of the modified membranes vs. pure chitosan from 128.5 °C in control to 120 °C in CS-PEG5. SEM results highlighted porous and anisotropic structure of crosslinked membranes. As the amount of PEG was increased, hydrophilicity of membranes was increased and water absorption increased up to a maximum of 67.34%. Permeation data showed that flux and salt rejection value of the modified membranes was increased up to a maximum of 80% and 40.4%, respectively. Modified films have antibacterial properties against Escherichia coli as compared to control membranes.

## 1. Introduction

Ocean and seas contain 97% of water present on earth. Out of 1% freshwater, about 0.007% is accessible while rest is present in the form of underground aquifers and surface estuaries [1]. Ten percent of the freshwater is consumed by human being for domestic purposes, while 70 % is used in agriculture and 20% in industry. The water left for domestic purpose is not sufficient, and more than 2.3 billion people are suffering from water shortage [2,3]. Overpopulation, industrialization, urbanization and excessive use of freshwater are the reasons for water shortage.

In the present era, one of the devastating problems being faced at a global level is water scarcity [4,5] and in order to meet freshwater demand, researches have been carried out. Desalination is a salt removal process and operated as a technological solution to overcome this problem [6,7]. The main purpose of desalination is to provide alternatives other than groundwater and freshwater for industrial, drinking and irrigation purposes [8]. Wastewater, brackish water and seawater are the main sources for desalination. Desalination can be divided into two types, which are thermal and membrane-based methods. The thermal process include multi-stage flash distillation, multiple effect distillation and vacuum distillation while reverse osmosis (RO), ultrafiltration (UF), microfiltration (MF) and nanofiltration (NF) fall into membrane classification [4,9]. A few of several available desalination methods have been used for freshwater production at an industrial level because of high energy demand and environmental impact.

Since the 1960’s, membrane-based methods have developed rapidly [10]. RO membrane technology has a share of 44% in the world desalination production capacity owing to its development over the past 40 years [4]. RO is defined as water desalination carried out by a pressure gradient through a semi-permeable membrane. The lower energy demand, maintenance and ease of operation are the main benefits of RO over the other desalination techniques, whereas its major disadvantage is membrane fouling which reduces membrane efficiency [11].

Various polymers have been used to prepare and enhance the performance of RO membranes and, preferably, economical and eco-friendly polymers are designated [12,13,14,15]. Chitosan is a polysaccharide and N-deacetylated derivative of chitin [16] synthesized by the alkaline deacetylation of chitin [17,18] which is the most abundant material after cellulose. Chitosan has several antibacterial, biocompatibility, biodegradability, antifouling and wound-healing properties. It has potential use in agriculture, beverages, medicine, gene delivery and water purification. It finds potential applications in heavy metals, organic matter and turbidity removal. Due to its hydrophilic, environmentally benign and film forming characteristics, it has been used in several separation techniques such as UF, NF and RO [19,20]. Being weakly basic, it is insoluble in alkaline and neutral medium but soluble at acidic pH [7]. Chitosan has a pK_a_ value of 6.2. It dissolves in acidic solutions with a pH value lower than 6.2. So, chitosan cannot dissolve in salt solutions with a pH of 6.59 [21]. Its amide group undergoes hydrolysis and protonation in acidic medium resulting in the hydrophilicity of membranes [22].

Polyethylene glycol (PEG-600) is a non-toxic polyether compound having a wide range of applications varying from industrial manufacturing to medicine, microbiology, gene therapy, lubricant, etc. As the molecular weight of PEG increased, water permeability (flux) of the membrane also increased [23]. A membrane of chitosan with PEG would impart hydrophilicity, thermal stability and resistance against fouling to the membrane [13,24]. Molecular weight and extent of crosslinking are the main factors which control the properties of the membranes. Silane cross-linkers are used to bind polymers resulting in enhanced properties of the modified membranes [25,26]. Silane cross-linkers impart strength, thermal stability and smoothness to the membranes [27]. They are also known to contribute to salt rejection of the resulting membranes. They have been used in a range of applications including drug release, separation of mixtures, water purification, etc., owing to their tremendous properties [28,29]. In this work, novel CS/PEG-600 polymer membranes for RO desalination were synthesized using APTES as a cross-linker which has not been developed earlier according to the best of our information [30,31,32]. The prepared membranes were characterized by scanning electron microscopy (SEM), Fourier transformed infra-red spectroscopy (FTIR), antibacterial testing, dynamic mechanical analysis and permeation properties in an RO plant. RO performance of membranes was measured in terms of permeate flux and percentage salt rejection, using a STERLITECH HP4750 stirred-cell-containing dead-end filtration system at 600 psi pressure after 6 h continuous operation.

## 2. Results and Discussion

### 2.1. Fourier Transform Infrared Spectroscopy

**Figure 1:**
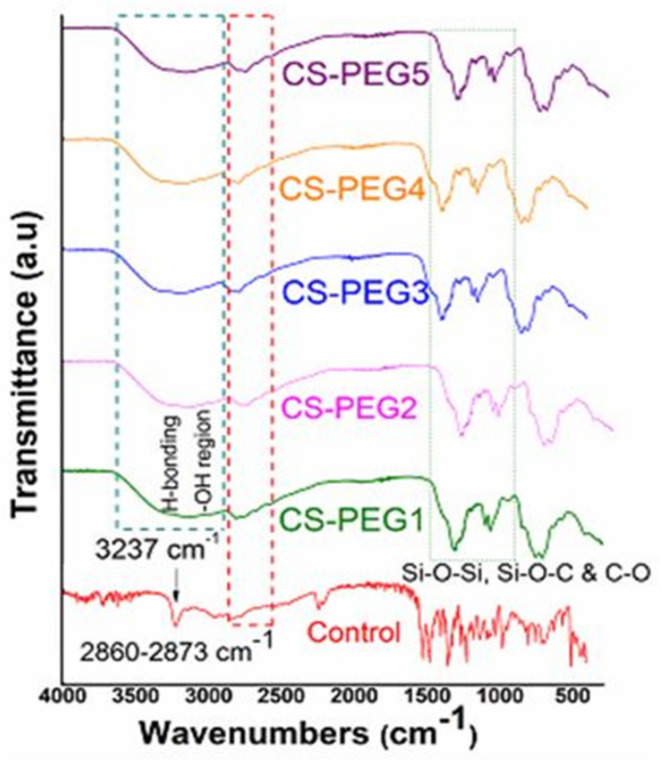
FTIR spectra of control and modified membranes revealing chemical interaction.

CS-PEG membranes may involve hydrogen bonding formation which can be complex in these membranes [33]. The confirmation of hydrogen as well as the covalent bonding among PEG, silanol and chitosan is indicated in Figure 1. The broadening of the −OH peak is observed at 3237 cm^−1^ in pure chitosan after membrane formation [34,35,36,37]. The peaks at 2873–2860 cm^−1^ in crosslinked membranes formed due to stretching vibrations (C–H vibrations) of PEG and chitosan [38]. In pure chitosan, N-acetyl glucosamine showed an absorption peak at 1320 cm^-1^. The bands formed at 1158 cm^−1^ in CS-PEG modified films are the result of stretching vibration (C-O vibration) [34,35]. The crosslinking of chitosan with PEG through reactions of -OH groups of PEG, Si-OH (silanol) chitosan are confirmed by the peaks formed in the 1192–1000 cm^-1^ region due to the development of Si-O-C and Si-O-Si groups [36].

### 2.2. Water Content Measurement

Hydrophilicity of the membranes is analyzed in terms of water content of control and modified membranes [39]. The increase in water content has been observed (Table 1), and the increase in concentration of PEG confirmed the increased hydrophilicity of the modified membranes [40]. It is mainly the hydrophilic nature of PEG-600 which caused water absorption into the crosslinked membrane. A significant role is played by this absorption in RO performance. The hydrophilicity of the control membrane is low as compared to the composite membranes. It is due to the reason that in control membrane PEG has not been added so water content of control membrane is 52 [6,41,42].

### 2.3. Reverse Osmosis Performance

#### 2.3.1. Flux

**Figure 2:**
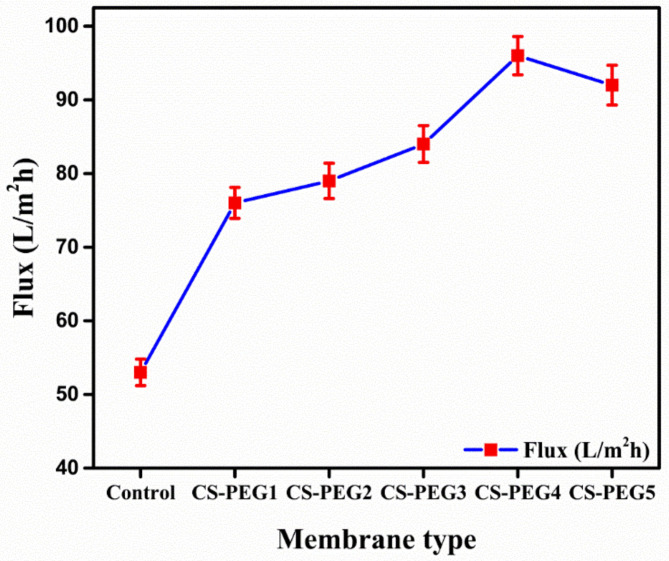
Variation in permeate flux with respect to variation in membrane composition.

Figure 2 shows the water flux of controlled as well as silane crosslinked membranes. The modified membranes presented enhanced flux values as compared to the control membrane. The transport within the membranes generally follows the solution diffusion model. Three steps are mainly involved in the transport process following this model, i.e., (1) sorption, (2) diffusion and (3) desorption [43]. Being hydrophilic, PEG imparts hydrophilicity to the crosslinked membrane and facilitates sorption of water at the membrane surface [44]. Acting as a porogen, it caused pore formation in the crosslinked membranes so water flux is increased [45]. CS-PEG4 showed maximum flux (96 ml/h.m^2^) owing to large amount of PEG 600 as shown in Figure 2. Furthermore, water flux of crosslinked membranes is increased by the addition of silica in the form of APTES but within certain concentration range [36]. Above that concentration limit, membrane fouling may lead to a decrease in flux as the time passed. A phenomena known as compaction is responsible for the decrease in flux with the passage of time. According to this phenomenon, polymeric chains undergo rearrangement under the influence of pressure. Resultantly, this decline in flux occurred due to alteration in membrane configuration followed by lowering in volume of porosity [15,46]. Aggregates of particles caused clogging of modified membranes resulting in the decrease in permeation flux [6,47].

#### 2.3.2. Salt Rejection

**Figure 3:**
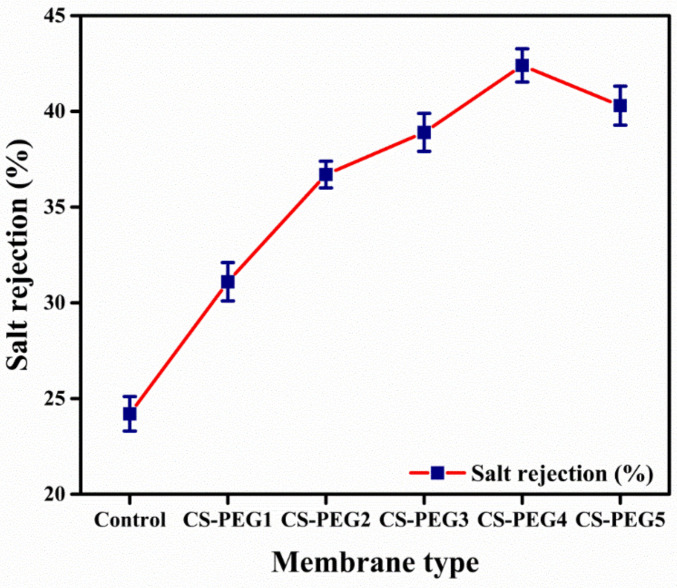
Variation in salt rejection with respect to variation in membrane composition.

Figure 3 shows the salt rejection of controlled as well as modified silane crosslinked membranes. The modified membranes exhibited improved salt rejection as compared to the control membrane. The salt rejection is increased from CS-PEG1 (31.1%) to CS-PEG4 (42.4%). PEG plays a role in permeation flux as well as salt rejection. In addition to its hydrophilic property, it enhanced the smoothness of the membrane, and facilitated salt rejection. In case of CS-PEG5, crosslinked membrane salt rejection capacity (40.3%) is compromised due to macro voids formation. These macro voids allowed salt to pass through it while water is passing. Consequently, water flux is raised but salt rejection is reduced [6,42]. In addition, Donnan effect promotes salt rejection due to electrostatic repulsion. According to Donnan effect, ions are excluded by columbic forces generated by the charged surface of the membrane [48,49]. However, salt rejection is affected by fouling in CS-PEG5, as is obvious from Figure 3 [36,41,50,51].

### 2.4. Antibacterial Activity

**Figure 4:**
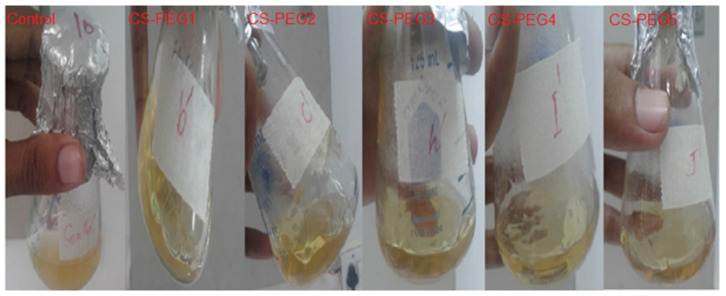
Influence of membrane composition on antibacterial activity of membranes.

The antibacterial activity is determined by the JIS L 1902-2002 method against *E. coli*. Turbidity is observed in the flask containing the control membrane which indicated the growth of bacteria. Observed optical density (OD) and bacterial killing ability (%) for control and modified membranes are reported in Table 1. OD and bacterial killing ability (%) values of all modified membranes designated that in these flasks, there is a negligible bacterial growth. One of the appropriate mechanisms of antibacterial activity is the electrostatic interaction of positively charged protonated amine (NH_3_^+^) groups of chitosan molecules and negatively charged bacterial cell membrane. These interactions disturbed the internal osmotic pressure of the bacterial cell, which ultimately inhibited the bacterial growth. According to a second proposed mechanism, chitosan penetrated into cell walls of bacteria and bound with their DNA, which prevented transcription of mRNA. The intermolecular hydrogen bonding of chitosan is reduced by the moieties from APTES and the −OH group of PEG. These groups kept chitosan molecules away from each which favored their solubility. So, modified membranes penetrated into bacterial cell easily and inhibited *E. coli* growth by preventing the transformation, as is obvious from Figure 4 [52,53]. Hence, the modified membranes inhibited the bacterial growth efficiently [15,54].

### 2.5. SEM Analysis

**Figure 5:**
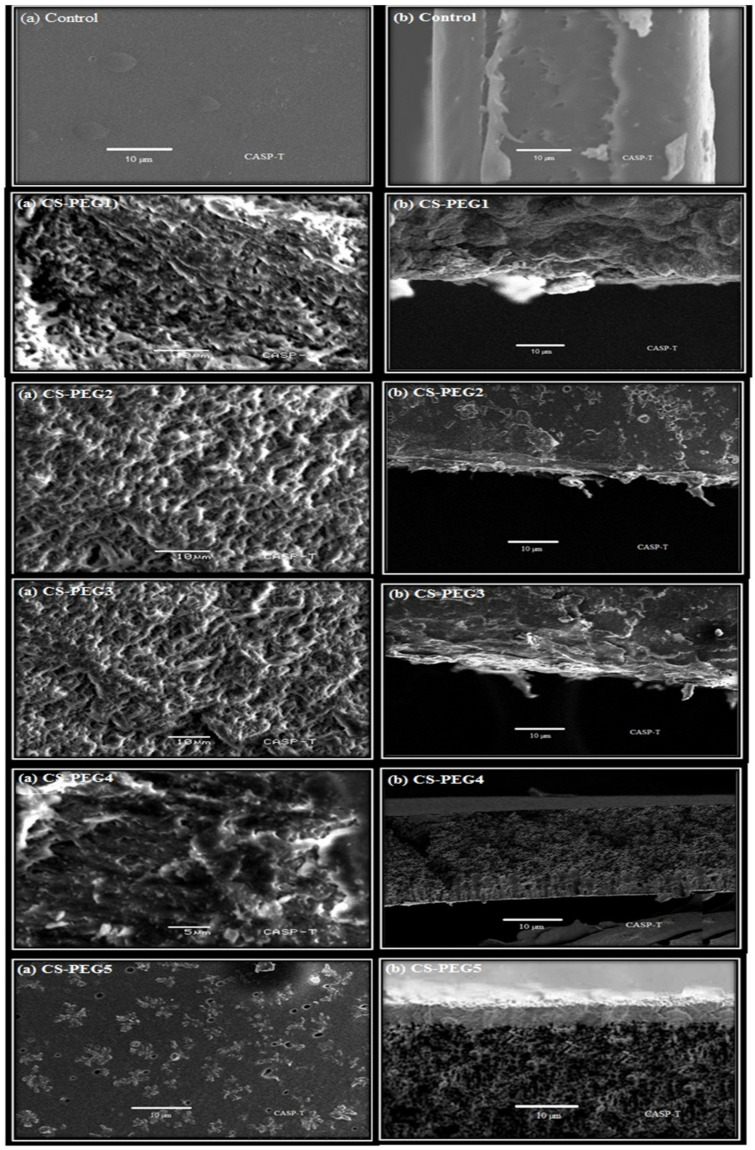
SEM images of surface and cross section of control and modified membranes. (**a**) shows surface views of control and modified membranes while (**b**) shows cross section view of membranes. SEM analysis facilitates the understanding of morphology of membranes which play vital role in salt rejection and permeation. The surface and cross sectional view of control membrane highlights the dense and nonporous micro structure of control membrane. Control membrane has this structure due to absence of PEG. CS-PEG membranes show porous structure as it can be seen in the figure below. PEG is known as porogen due to its pore generating property. A direct relationship is observed between the amount of PEG and pore size. Due to this fact, an increase in amount of PEG leads to an increase in pore size of CS-PEG membranes.APTES being a crosslinker is involved in binding of PEG with the matrix through covalent bonding. Resultantly, it results in formation of modified CS-PEG membranes as obvious from given figure. This bonding has been shown in the proposed reaction mechanism as well.

To understand the basics of permeation and salt rejection mechanism of membrane, surface analysis of the membrane is essential [10]. SEM images of surface as well as cross-sections of control and modified membranes are given in (Figure 5a,b), respectively. In case of control membranes, a dense and uniform microstructure devoid of pores is observed. However, CS-PEG membranes exhibited a porous structure and the amount of PEG has a direct influence on the pore size [55]. The higher the amount of PEG, the larger the pore size and eventually more porous membrane is formed. This porosity induced in modified membranes by adding PEG revealed the fact that there is a weak interaction between a porogen and chitosan [56].

### 2.6. Dynamic Mechanical Analysis

**Figure 6:**
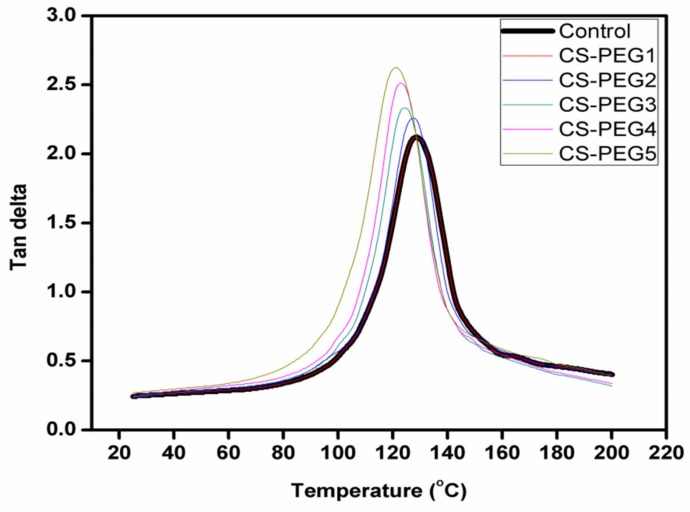
A comparative analysis of variation in tan delta of control and modified membranes with change in temperature.

Being more sensitive, DMA has several advantages over differential scanning calorimetry as it allows accurate verification of T_g_, in addition to secondary transitions of polymer segments [57,58]. In DMA, T_g_ is defined as that value of temperature where tan δ shows maximum value or that value where α transitions occur. DMA scans of control and crosslinked membranes can be seen in Figure 6 and T_g_ values are given in Table 2. It is obvious that variation in concentration of PEG greatly influenced T_g_ of the crosslinked membrane [59]. At 0.1 mL of PEG, being a low concentration, same values of T_g_ are observed in DMA scans of control and CS-PEG1. However, a decrease in T_g_ value of crosslinked membranes is observed with an increase in PEG content above 0.1 mL (Table 2). It is due to the fact that PEG, being a porogen, generated free volume in microstructure of chitosan at high concentration.

Damping capacity of the control and crosslinked membranes is also measured with the help of T_g_. The decrease in T_g_ of crosslinked membranes with respect to increase in concentration of APTES within the chitosan matrix is quite prominent at high concentration of APTES because APTES restricted the motion of polymeric segments of chitosan, and the following trend is observed in damping capability of control and crosslinked membranes, as is obvious from Figure 6.

CS-PEG5 > CS-PEG4 > CS-PEG3 > CS-PEG2 > CS-PEG1 ≥ Control [60,61].

**Figure 7:**
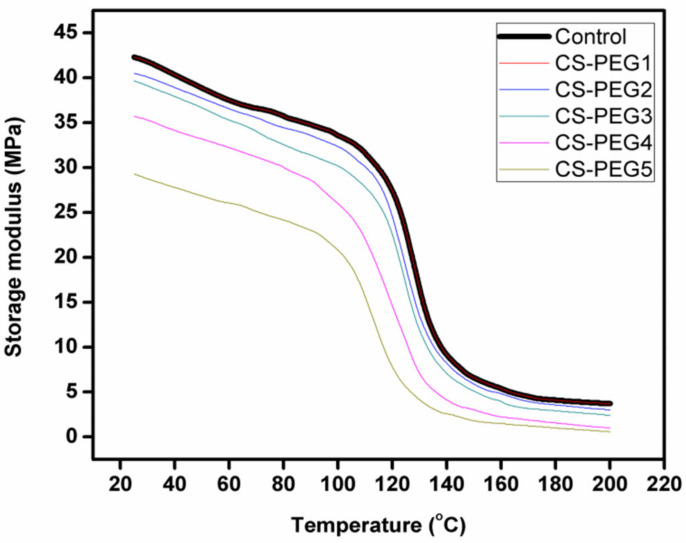
Temperature dependence of storage modulus in control and modified membranes.

Figure 7 shows that storage moduli of control and crosslinked membranes decreased at a specific value of temperature. At this temperature, the glassy state transformed into a rubbery state. The shift in values of tan δ and storage modulus to lower values with the increase in concentration of PEG is typically observed in a plasticizer [61].

The introduction of APTES into chitosan increased rigidity in its chains. That is why a direct relation has been observed between E’ value and concentration of APTES in crosslinked membranes. It is the reason that decrease in E’ value of crosslinked membranes is observed as the concentration of APTES decreased form CS-PEG1 to CS-PEG5 [60].

## 3. Experimental

### 3.1. Materials

Analytical grade chitosan (CS) ((M.W = 955434.8 g/mol), (viscosity = 1000 cp) and degree of deacetylation = 90%)) was purchased from Biolog (GmbH), Germany. PEG-600 (M.W = 600 g/mol) and formic acid (% purity = 88–91%) were bought from MERCK. APTES of 99% purity (M.W = 570.630 g/mol) was also imported from MERCK. All chemicals were of analytical grade used as received.

### 3.2. Membrane Preparation

#### 3.2.1. Preparation of Control Membrane

Chitosan (1.5 g) was dissolved in formic acid solution (3%) and stirred constantly for 5–6 h at 60 °C. The subsequent polymer solution was evaporated and poured on a glass plate. The obtained film (control membrane) was dried at room temperature and removed by using a doctor’s blade.

#### 3.2.2. Preparation of Modified Membranes

**Figure 8:**
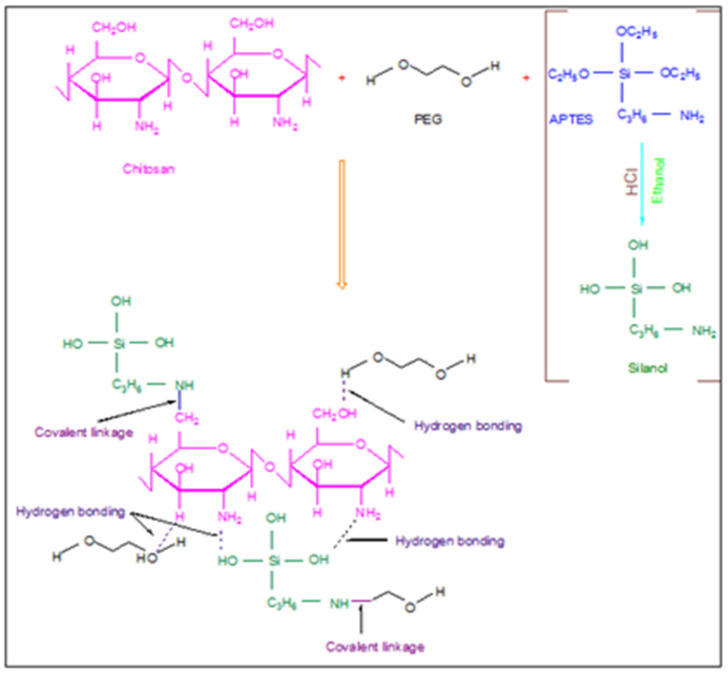
Proposed mechanism of interaction of chitosan, polyethylene glycol (PEG) and 3-aminopropyltriethoxysilane (APTES) resulting in membrane formation.

Chitosan (1.5 g) was dissolved in formic acid solution (3%) and stirred constantly for 5–6 h at 60 °C. A specific amount (Table 3) of PEG was added into chitosan solution and blended for half an hour. Then, the respective concentration (Table 3) of APTES (dissolved in 3 mL of ethanol) as a cross-linker was poured dropwise into stirred cell containing dead end filtration system the chitosan-PEG blend and constantly stirred for 5–6 h at 60 °C. Five compositions (CS-PEG1, CS-PEG2, CS-PEG3, CS-PEG4 and CS-PEG5) were prepared by using varying concentrations of PEG and APTES as shown in Table 3. The resultant solution was evaporated and poured on glass plates. The prepared membranes were dried at room temperature and removed by using a doctor’s blade. A proposed reaction mechanism for membrane formation is given in Figure 8.

## 4. Characterization

Several techniques were utilized in order to characterize the prepared polymer membranes.

### 4.1. Fourier Transform Infrared Spectroscopy

A Perkin Elmer RX 1 FTIR spectrophotometer was used to record FTIR spectra of samples. The spectra were recorded in transmission mode and wavenumber range was adjusted from 4000–400 cm^−1^. Air was used as background, 32 scans were carried out for a spectrum and resolution was adjusted at 2 cm^−1^.

### 4.2. Percentage Water Content

Equal weights of each sample (25 mg) were taken in separate petri dishes. The dried samples were immersed in water (100 mL) for 24 h. The blotting paper was used to wipe out extra water from membrane samples after 24 h and weights of wet samples were measured. These membrane samples were then subjected to drying for 48 h at 70 °C under vacuum in an oven, and weights of dried samples were determined. The water content (WC %) was calculated using Equation (1) [62].

(1) $$\mathrm{WC}\%=\frac{\mathrm{WS}-\mathrm{DS}}{\mathrm{WS}}\times 100$$

where, WC stands for water content, WS stands for weight of wet sample and DS stands for weight of dried sample.

### 4.3. Reverse Osmosis Performance

**Figure 9:**
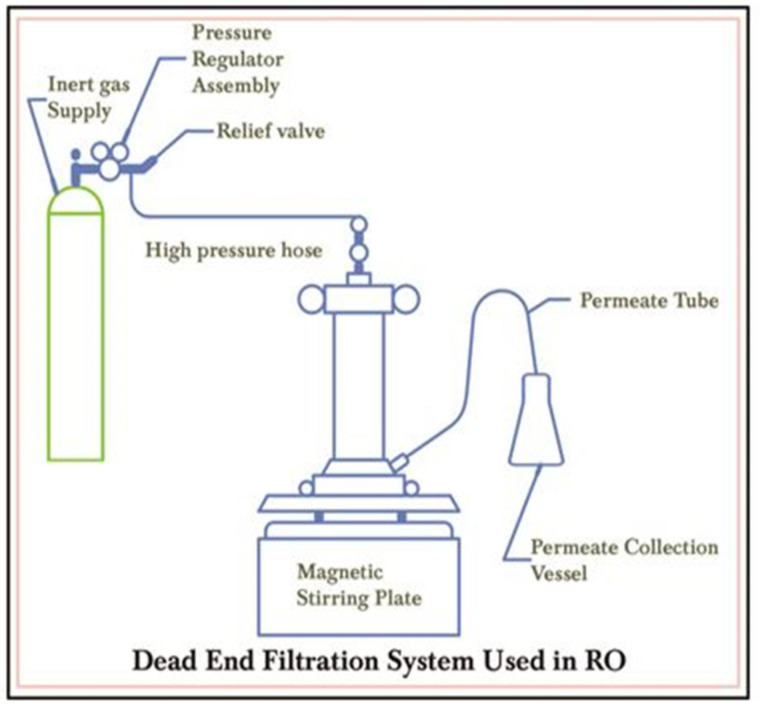
Filtration system used to characterize salt rejection and permeate flux of membranes in reverse osmosis (RO).

Membranes were characterized in terms of salt rejection (%) and permeate flux by using a STERLITECH HP4750 stirred-cell-comprising dead-end filtration system as shown in Figure 9. at 600 psi pressure after 6 h continuous operation [63]. An amount of 3.28% solution of NaCl (pH=6.59) was prepared to carry out the RO performance test.

#### 4.3.1. Percentage of Salt Rejection

Measurement of the capability of a prepared membrane to reject the salt content of a water sample was calculated by using Equation (2).

(2) $$\mathrm{Percentage}\;\mathrm{salt}\;\mathrm{rejection}=\left[1-\frac{\mathrm{Cp}}{\mathrm{Cf}}\right]\times 100$$

where, C_p_ stands for permeate conductance and C_f_ stands for feed conductance.

#### 4.3.2. Permeate Flux

The flow rate of the prepared membrane was assessed by permeate flux evaluation. It was measured by Equation (3) [15].

(3) $$J=\;\frac{Q}{t\times A}$$

where, J stands for membrane flux (mL/h.m^2^), t stands for time, Q stands for permeate volume (mL) and A stands for area (m^2^).

### 4.4. Antibacterial Analysis

This test was performed using the JIS L 1902-2002 method against *Escherichia coli*. Six Erlenmeyer flasks of 250 mL volume were used to prepare 30 mL of broth. All the flasks were autoclaved at 15 psi pressure at 125 °C for 30 min. After autoclaving, 100 µL of DH5 alpha *E. coli* strain was inoculated in the Erlenmeyer flasks. *E. coli* strains were divided into six categories: BC, BM_1_, BM_2_, BM_3_, BM_4_ and BM_5_. Membranes of different composition were added to BM stains and unmodified membrane was added to BC strain. All flasks were incubated at 35 °C for 24 h after which optical density (OD) at 600 nm wavelength was recorded by using a spectrophotometer. The modified French and Lee (2012) method was used for quantitative determination of antibacterial properties of the prepared membranes. Bacterial killing ability (%) was calculated as one, minus mean absorbance of each BM sample, divided by the mean absorbance of the positive control BC and multiplied by 100 [64].

### 4.5. Scanning Electron Microscopy

A Jeol field emission electron microscope (JSM-6480) was used to characterize the morphology of membranes. The technique mainly involves trimming the membrane samples into small pieces that were then subjected to electron beams in a vacuum chamber. It was followed by the analyses of images under varying resolutions.

### 4.6. Dynamic Mechanical Analysis

DMA 2980 apparatus from TA instruments was used to assess the glass transition temperature (T_g_), storage modulus (E’) and damping factor (tan δ) of the membrane samples. The temperature for this test was maintained from 25–200 °C at the rate of 3 °C/min and 10 µm was the oscillation amplitude. This test was performed at 1 µm frequency.

## 5. Conclusions

Chitosan/PEG-600 membranes crosslinked with APTES for RO desalination have been successfully prepared and characterized. The results confirmed that as the amount of PEG-600 was increased, hydrophilicity of crosslinked membranes was also increased. PEG-600, being hydrophilic, increased water content of membranes. With the increase in concentration of PEG-600, water flux (96mL/h.m^2^) and salt rejection (42.4%) of the modified membranes was increased but up to a certain concentration (CS-PEG 4). OD values of modified membranes revealed better antibacterial activity than control membrane. SEM analysis showed that CS-PEG modified membranes exhibited a porous structure, and the amount of PEG has a direct influence on the pore size. T_g_ as well as storage modulus of control and modified membranes were evaluated by DMA. This analysis revealed a reduction in T_g_ as well as storage modulus of modified films in response to variation in concentration of APTES and PEG.

## Figures and Tables

**Table 1:** Water content (%), antibacterial activity and bacterial killing ability (%).

Sr. #	Membrane Type	Water Content (%)	Standard Deviation	Optical Density (OD) at 600 nm	Standard Deviation	Bacterial Killing Ability (%)	Standard deviation
1	Control	52	16.10	0.532	0.04	-	-
2	CS-PEG1	90.14	0.94	0.512	0.03	91.7	9.57
3	CS-PEG2	93.56	2.47	0.488	0.02	96.2	7.32
4	CS-PEG3	96.02	3.57	0.461	0.01	101.3	4.77
5	CS-PEG4	97.71	4.33	0.322	0.04	127.4	8.28
6	CS-PEG5	98.73	4.78	0.268	0.07	137.6	13.38

**Table 2:** Glass transition temperature (T_g_) value of control and modified membranes.

Sr. #	Membrane Type	T_g_
1	Control	128.5
2	CS-PEG1	128.5
3	CS-PEG2	127.6
4	CS-PEG3	124.5
5	CS-PEG4	122.9
6	CS-PEG5	120

**Table 3:** Different compositions of prepared membranes.

Sr. #	Compositions	Amount of Chitosan (g)	Volume of APTES (mL)	Volume of PEG (mL)
1	Control	1.5	0	0
2	CS-PEG1	1.5	0.9	0.1
3	CS-PEG2	1.5	0.7	0.3
4	CS-PEG3	1.5	0.5	0.5
5	CS-PEG4	1.5	0.3	0.7
6	CS-PEG5	1.5	0.1	0.9

## Data Availability

The raw/processed data required to reproduce these findings cannot be shared at this time due to legal or ethical reasons.
